#  Agent Based Simulation of Sale and Manufacturing Agents Acting Across a Pharmaceutical Supply Chain 

**Published:** 2018

**Authors:** Narges Pourghahreman, Ali Rajabzadeh Ghatari, Asiye Moosivand

**Affiliations:** a Department of Information Technology Management, Faculty of Management and Economics, Science and Research Branch, Islamic Azad University, Tehran, Iran.; b Department of Management, Faculty of Management and Economic, Tarbiat Modares University, Tehran, Iran.; c Department of Pharmacoeconomy and Administrative Pharmacy, School of Pharmacy, Shahid Beheshti University of Medical Science, Tehran, Iran.

**Keywords:** Agent based simulation, Case study, Mathematical modeling, Regression analysis, Pharmaceutical industry, Iran

## Abstract

Agent based modeling and simulation consider the behavior of agents acting in a system. The agents’ interactions result in changing the agent’s behavior, the whole agent based system, and its environment. In this study, the manufacturing, sale, and receiving orders behaviors pertinent to manufacturing and sale agents acting across a pharmaceutical supply chain of an Iranian manufacturing medicine (as a case) are simulated. The departments related to these two agents have some problems affecting the entire supply chain; the results are interpreted based on agile, lean, and green paradigms. During the research, three medicines were selected and the related data were gathered. Then, mathematical modeling and regression analysis (in some parts) were applied. Next, a computer model was composed on matrix library environment (MATLAB). Finally, four scenarios were simulated. According to the information resulted from simulating the first scenario, none of supply chains pertaining to each medicine is agile. Based on the findings of simulating the second scenario, decreasing waste leads the non-antibiotic medicine supply chain to move toward the lean and green paradigms more. According to the third scenario, although more order requests can be fulfilled by increasing production capacity, the supply chain will not become agile. The last scenario’s goal is checking the possibility of receiving 145000 units’ orders for the non-antibiotic medicine which should be prepared during next 70 days while the company confronts lack of raw material and the suppliers are domestic; based on the results, the order will be rejected due to lack of time.

## Introduction

These days, a non-stop changing world and globalization are matters influencing many distinct scopes of which business is one. To be progressive, business owners cannot neglect the important role of having supply chains (SC) and managing them in the best manner. Adjusting the business situation with these facts is essential if they want to improve their business. Globalization causes the nature of competition to be altered from company versus company to supply chain versus supply chain (1). The managerial ability to integrate and coordinate the complex network of business relationship across supply chain members is required for final victory of firms (2). These are some evidences indicating the momentous nature of supply chains. A rich playground is provided by supply chain scope for researchers to do sophisticated studies (3).

Agent technology is a new paradigm that can be exploited in many industries (4). The consideration for linking systems, independent operations, representation of profits and making decisions among individuals or systems’ interactions result in the establishment of the agents and at extension the multiagent systems (5). Multiagent systems consist of heterogeneous agents interacting with each other. Additionally, a supply chain can include manifold stakeholders having their own suppliers, manufacturers, distributors, third party logistics providers, retailers, and customers (6). All of these reasons represent supply chain systems are complex. Agents are applied for designing or simulating intricate systems (3). Consequently, applying agent technology is appropriate to supply chain scope. Any elements of supply chain like suppliers, manufacturers, distributors, and customers can be considered as agents because they possess the goal-directed behavior, interact with the other elements, and respond to the environment while they are autonomous.

Keeping in view the both importance of supplying medicines helping to promote the health situation in societies and tremendous impacts of pharmaceutical industry on the environment, this study was done in field of the mentioned industry. It is significant to investigate how elements like manufacturing and sale agents involving across the pharmaceutical supply chain have impact on the performance of each other, the entire chain, and on the environment too. As a result, managers can make proper decisions based on more enough detailed information. Investigating the role of each supply chain part based on agent based simulation approach is approximately new in Iran. 

The research was done in an Iranian company as a case for study manufacturing three groups of medicines: antibiotic, non-antibiotic, and semi-solid medicines. ISO 14001, ISO 9001, and good manufacturing practice (GMP) have been implemented in the company. This study focuses on sale and manufacturing agents considered as agents playing a role across the manufacturer layer of supply chain to investigate and simulate some behaviors of them. 

*Defining some concepts*

Before explaining the application of multiagent systems in supply chain area, it would be proper to define concepts like supply chain, modeling, and agent technology.

Supply Chain: A supply chain (SC) is comprised of suppliers, factories, warehouses, distribution centers, and retailers integrated in order to 1) prepare raw material and parts 2) transform these raw material and parts in to final products 3) add value to the products 4) distribute goods to the retailers or customers 5) comfort information exchange across supply chain elements. Improving operational efficiency, profitability, and promoting competitive position of a company is the main aim of possessing supply chain (7). Supply chain management (SCM) can be defined as the strategic, tactical, and operational decision making process that optimizes SC performance (8).

Agility: As mentioned before, nowadays we face a business world where change is its inherent specification; agility is a paradigm empowering a firm to respond to market fluctuations and other uncertainties in a timely and effective manner. As a result, firms can establish a superior competitive position (9). An agile supply chain is derived from integrating the supply chain’s alertness to changes in order to utilize resources in responding (proactively/reactively) to such changes, all in a timely and flexible manner (10).

Lean paradigm: The lean approach focuses on the elimination of waste for increasing actual values to fulfill customers› needs and to maintain profits. In other words, it is about doing more with less. Everything or each operation and activity which is non-value adding can be considered as waste; processes improvement is the waste elimination consequence. The lean supply chain is a strategy based on cost reduction and flexibility emphasizing on processes improvement, through the reduction or elimination of the all non-value adding operations which are wastes (11, 12).

Green paradigm: The philosophy of the green paradigm is to achieve profit and market share objectives by reducing environmental risks while amending ecological efficiency of the enterprises and their partners (13, 14). Green supply chain management territory includes a wide spectrum of all the supply chain activities, from green purchasing to integrate lifecycle management, through to manufacturer, customer, and closing the loop with reverse logistics (14).

Agent and multiagent systems: The term “agent” explains a hardware or (more usually) software-based computer system. Autonomy, social ability, reactivity, and pro-activeness are four characteristics of each agent (15). As in every technology, the agents have a variety of special skills thus they are applicable for distributed, unstructured and decentralized architectures which are complicated due to changes. Agents communicate, collaborate, coordinate and negotiate in a system designed and implemented as a multiagent system (5). Multiagent technology could be an appropriate option to model and simulate the collaboration mechanism and processes of a system (1).

*Multiagent systems in supply chain*

a supply chain is a set of heterogeneous production subsystems interacting in wide dynamic and virtual coalitions, where each production subsystem has its individual goals, while satisfying both local and external constraints. Besides, autonomy, social ability, reactivity, and pro-activeness are four characteristics of the distributed manufacturing units. Agent technology and particularly multiagent systems have been created for dealing with such characteristics. Moreover, multiagent systems provide a way for realizing systems that are decentralized rather centralized, emergent rather planned, and concurrent rather sequential; this is what characterizes supply chain systems generally. It is the logic of choosing such technology in supply chain management area (16). Lots of researches have been done in field of agent technology application in supply chain. Table 1 contains some of them.

**Table 1:** A brief review to some researches which have been done in field of agent technology application in supply chain since 1991

**Year**	**Brief explanation**	**Researcher (s)**
1998	A simulation frame work using agent technology is provided for developing customized supply chain models.	Swaminathan, Smith and Sadeh (17)
1999	A collaborative agent system architecture and an infrastructure for collaborative agent systems are proposed. The architecture is general for an internet-based multiagent system and is very suitable for managing complex supply chains in large manufacturing enterprises.	Shen, Ulieru, Norrie and Kremer (18)
2000	The researchers developed a framework of agent-based electronic markets (e-markets) to simulate the dynamic transaction situations from subcontractors.	Kim, Boyd, Paulson, Charles and Petrie (19)
2000	An agent-based software system is proposed for assisting SCM decision making.	Pathak, Nordstrom and Kurokawa (20)
2000	A Supply-Chain Web Centric System, called the SC-Web-CS, which could provide different domain entities such as services, providers, transports, ordering, manufacturers, customers, distributors, and retailers are developed.	Wu, Cobzaruo, Ulieru and Norrie (21)
2002	The study focuses on how application of multiagent systems for SCM can amend the inter-company data exchange, procurement, and coordination of production in mass customization.	Turowski (22)
2003	Agent strategies in a supply chain model are formulated according to the virtual market concept with multiple agents.	Kaihara (23)
2005	A framework of an e-supply chain to provide an intelligent e-marketplace with multiple agents is offered by this research.	Singh, Salam and Iyer (24)
2007	An agent modeling framework for the modeling and simulation of supply chains is presented to facilitate their management. It is manifested how the framework can be applied to a case of customer-centric supply chain from the golf club industry.	Labarthe, Espinasse, Ferrarini and Montreuil (25)
2010	The paper addresses a new methodological framework which permits modeling and simulation of supply chain organizational aspects.	Mustapha *et al. *(3)
2011	An agent-based simulation framework for supply chain (SC) planning is introduced. The notion of normative agent is applied on this research.	Ferreira and Borenstein (26)
2012	The research objective is to tackle the issues under which agents can coexist in a competitive environment. Furthermore, the supply chain management trading interaction among agents is specified by using an optimization approach based on a genetic algorithm (GA), clustering and fuzzy logic (FL).	Djennas, Benbouziane and Djennas (27)
2012	This article gives an introduction to agent based Modeling and simulation (ABMS). It addresses the basic concept of ABMS, focusing on its generative and bottom-up nature, its advantages as well as its pitfalls	Klügl and Bazzan (28)

## Experimental

*Research methodology*

Preparing pharmaceutical required materials, manufacturing, and distributing medicines are significant matters in societies. Unfortunately, the pharmaceutical industry in Iran faces hardships originated from many reasons; sometimes some problems exist in the companies causing delays in supplying required medicines. All these reasons influence the performance of each element involving in pharmaceutical supply chain. Furthermore, changes occurred on the performance of each element affect the performance of the other ones and the whole of supply chain too because they interact with each other. These interactions encouraged us to choose agent based simulation approach. According to this approach, we can comprehend complex systems and remark that simple and also complex phenomena can be the result of interactions between autonomous and independent entities like agents operating within communities having different interaction modes (29). It is innovative because you can investigate the changes occurring in the behavior of a specific agent as a result of changes happening in other agents’ behavior or environment. For example, if manufacturing agent could produce more quantities, the sale agent would be able to accept more requests and the lost sales would be reduced. The aim of this study is simulating the manufacturing behavior, sale, and receiving order behaviors pertinent to manufacturing and sale agents acting across a pharmaceutical supply chain of an Iranian manufacturing medicine as a case study. These two agents are considered because after interviews done with the company’s chief executive manager and strategic planning manager in order to get general information of the supply chain, it was founded that the sale and manufacturing departments have some problems affecting the entire supply chain unfavorably. Additionally, three medicines which each of them are related to each group of medicines produced by the company were selected by the chief executive mangers in order to be studied. The research has been done in two main phases: 1) modeling and 2) simulation.

To accomplish the first phase, mangers of those two related departments were interviewed in order to gather the general data of their performance. After that, the information of daily quantities related to produced medicines were gathered. Then, the mathematical and computer modeling were done. In some parts, regression modeling was used while in the other parts the system was modeled mathematically based on information gathered. More information is described about regression and modeling phase during the next parts. 

The term computer simulation is pertinent to the application of a computational model to improve the understanding of a system›s behavior and/or to evaluate strategies for its operation, in explanatory or predictive schemes (29). In the simulation phase, the computer simulation was done and the results were analyzed based on lean, agile, and green paradigm. 

*Modeling phase*

A model represents the construction and working of some systems. A model is similar to the considered system but it is not as complex as it (30). As it was mentioned above, regression modeling is a way done during some parts of the research. This method is a mathematical approach describing a process in terms of a set of associated variables. The values of one variable frequently depend on the levels of several others (31). The single linear regression model and its parameters’ formulas are mentioned in the following (32):

$\mu \left(y|x\right)=\alpha +\mathrm{\beta x}$

(1)

(2) $$\overset{\text{\textasciicircum{}}}{\beta }=\frac{n(\sum_{i=1}^{n}x_{i}y_{i})-(\sum_{i=1}^{n}x_{i})(\sum_{i=1}^{n}y_{i})}{n(\sum_{i=1}^{n}x_{i}^{2})-{(\sum_{i=1}^{n}x_{i})}^{2}}$$

$\overset{\text{\textasciicircum{}}}{a}=\frac{\sum_{i}^{n}=y_{i-}\overset{\text{\textasciicircum{}}}{\mathrm{\beta .}}(\sum_{i}^{n}=1x_{i})}{n}$

 (3)

Karl Pearson developed a quantity called linear correlation coefficient measuring the strength and the direction of a linear relationship between two variables. Pearson’s r can range from -1 to 1. An r of -1 indicates a perfect negative linear relationship between variables, an r of 0 indicates no linear relationship between variables, and an r of 1 indicates a perfect positive linear relationship between variables. With real data, you would not expect to get values of r of exactly -1, 0, or 1. The mathematical formula for computing r is (33):

(4) $$r=\frac{\sum_{i=1}^{n}\left(x_{i}-\bar{x}\right)(y_{i}-\bar{y})}{\sqrt[{2}]{\sum_{i=1}^{n}{\left(x_{i}-\bar{x}\right)}^{2}\sqrt[{2}]{\sum_{i=1}^{n}{\left(y_{i}-\bar{y}\right)}^{2}}}}$$

There is another quantity known as coefficient of determination (r^2^) calculated based on correlation coefficient and defined as the proportion of variance explained by the regression model applied as a measure of success of predicting the dependent variable from the independent variables. It is applied in classical regression analysis; in fact, it is indicating how well the regression line represents the data (34).

*Mathematical modeling for sale agent*

In this company, the sale manager provides a report related to periods of every three months containing the information of all orders received and all medicines sold during the periods as well as the calculated quantities which are equivalent to daily received orders and medicines sold. To model cumulative quantities of received orders (Rx), medicines sold (Sx), and lost sales (LSx) for each medicine during t days, the mentioned information was gathered and the cumulative quantities were calculated. The models are mentioned on Table 2.

**Table 2:** The mathematical model pertinent to received orders (Rx), sold medicines (Sx), and lost sale (LSx).

	**The equation of received orders (Rx)**	**The equation of sold medicines (Sx)**	**The equation of lost sale (LSx)**
Medicine related to non- antibiotic group	Rn=19424×t	Sn=15124×t	LSn=4300×t
Medicine related to antibiotic group	Ra=12582×t	Sa=10798×t	LSa=1784×t
Medicine related to semi-solid group	Rs=15995×T	Ss=10824×T	LSs=5171×T

*Mathematical modeling for manufacturing agents*

**Figure 1:**
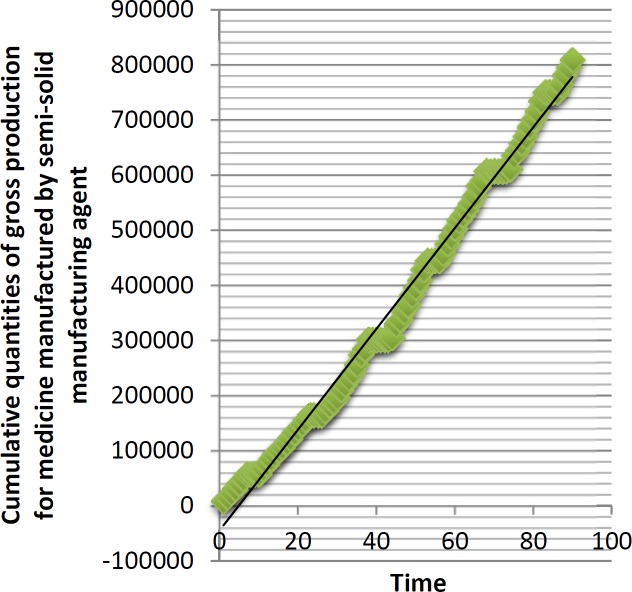
Cumulative quantities of gross production for medicine manufactured by semi-solid manufacturing agent.

To model the production capacity of manufacturing agents mathematically, the daily production information of each agent were gathered during 90 days and the cumulative quantities of gross production for each manufacturing agent were calculated. After that, the pertinent charts were drawn. Figure 1 depicts an example of them. Then, regression modeling was applied. Table 3 includes the result of the modeling. GPx is the abbreviation of cumulative gross production of the medicine x. Because the coefficient of determination (r^2^) for each equivalent is near to 1, all equivalents model the related behaviors in the best manner.

**Table 3:** The regression models of cumulative gross production for each manufacturing agent

	**The regression model of cumulative gross production (GPx)**	**r**	**r** ^2^
The non-antibiotic agent	GPn=15587.760×t-19717.660	0.994	0.988
Antibiotic manufacturing agent	GPa=12842.905×t-97596.462	0.992	0.984
Semi-solid manufacturing agent	GPs= 9144.060×t-44710.494	0.995	0.99

 To model manufacturing agents’ producing waste behavior two parameters (mx) and (nx) calculated by the company management representative for each medicine considered; (mx) is portion of waste produced daily, and (nx) is portion of daily net production too. Therefore,the cumulative produced waste (Wx) and the cumulative net production of x (NPx) during t days can be calculated as follow:

Wx=mx×GPx                                                         (5)

NPx=nx×GPx                                                         (6)

Nx=1-mx                                                         (7)

Table 4 contains the Wx and NPx models related to each considered medicine. 

**Table 4:** The mathematical models related to waste produce by manufacturing agents and their net production capacity

	**Wx**	**NPx**
Mathematical model of non-antibiotic manufacturing agent’s behavior	Wn = 0.005600682 × GPn	NPn = 0.994399318 × GPn
mathematical model of antibiotic manufacturing agent’s behavior	Wa = 0.003054849 × GPa	NPa = 0.996945 × GPa
Mathematical model of semi-solid Manufacturing agent’s behavior	Ws = 0.0044090946 × GPs	NPs = 0.995909054 × GPs

*Computer modeling*

The simulation program was composed on MATLAB based on the information gathered. Simulation time (T), order quantity (oq), portion of waste pertinent to each medicine produced daily (mx), and portion of net production relevant to each medicine (nx), as well as coefficient of production capacity (A) are considered as parameters can be adjusted by users. In addition, users can determine that the material is provided by foreign or domestic suppliers. Based on the interview done with the company chief executive manager, when needed material is provided by domestic suppliers, lead time is 90 days; it is considered 180 days when suppliers are foreign. The lead time is applied for comparing total needed time for manufacturing received orders (nt) and Simulation time. For computer modeling, the mathematical models were used. For example, you can follow the logic behind a part of computer modeling when the aim is to investigate production feasibility for the specified order quantities during a determined period by means of simulation.

$\left\{\begin{array}{c} \mathrm{If T}<\mathrm{lead time the order will be rejected}\\ \mathrm{f T}>\mathrm{lead time the nt should be rejected} \end{array}\right.$

(8)

The general format for the formula of all GPx is mentioned in following:

 (9) GPx=a+b×t

Based on the formula, the required time for process of producing received orders (pt) is formulated as follow:

oq=a+b×pt

(10)

(11) pt=oq-a b

$\mathrm{bt}=\mathrm{pt}+\mathrm{lead time}\Rightarrow \left\{\begin{array}{c} \mathrm{nt}=\mathrm{pt}+90\mathrm{Domestic suppliers}\\ \mathrm{nt}=\mathrm{pt}+180\mathrm{Foreign}/\mathrm{Foreign and domestic suppliers} \end{array}\right.$

(12)

$\left\{\begin{array}{c} \mathrm{If nt}>\mathrm{T 0 the order will be rejected due to lack of time}\\ \mathrm{f nt}<\mathrm{T the orders can be manufactured if there are not any other limitations} \end{array}\right.$

(13)

During writing the program, the codes were supervised, tested, and confirmed by two experts who are university professors teaching simulation courses and having at least three years experience of teaching MATLAB too. Finally, when the simulation program was written entirely, it was tested by experts several times; the results were based on the reality. For example, when the program was run for the antibiotic medicine, we came in conclusion the supply chain is not agile according to charts and numeric data derived. In the real world, the company’s supply chain pertinent to each medicine is not agile too.

*Simulation phase*

Simulation is the imitation of a real world process or system; gathering artificial history of a system reached by simulation and observing them propel us to apprehend operating characteristic of the real system modeled (35). Simulation is applied in different situation. For example, it is used before altering an existing system or building a new one to reduce the chances of failure, meet specifications, eliminate unforeseen bottlenecks, prevent under or over-utilization of resources, and optimize system performance (30). Many scenarios can be considered to be simulated; in this paper, four scenarios are considered for simulating the supply chain. They are mentioned during next parts. 

*Scenario 1*

**Figure 2:**
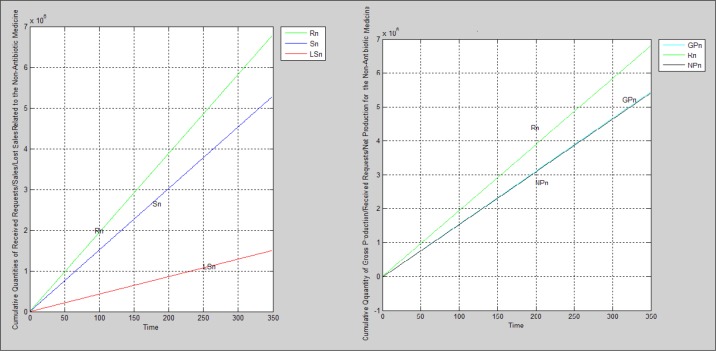
The non-antibiotic medicine’s charts resulted from running the simulation program based on scenario 1

**Figure 3:**
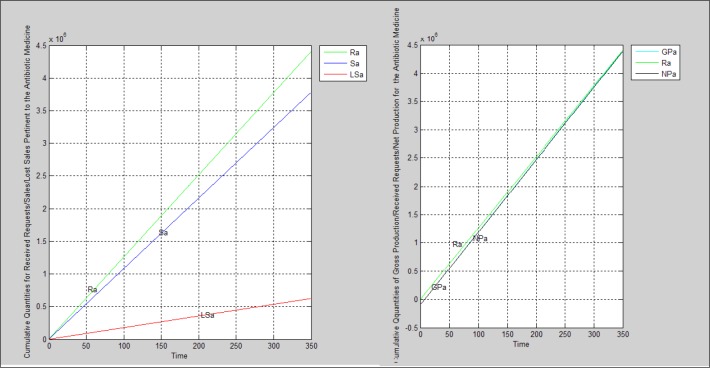
The antibiotic medicine’s charts resulted from running the simulation program based on scenario 1

**Figure 4:**
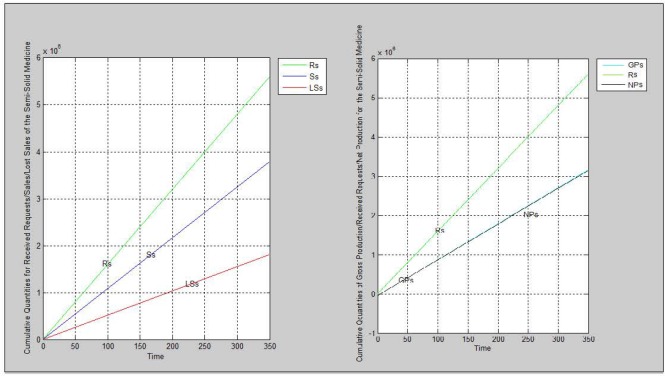
The semi-solid medicine’s charts resulted from running the simulation program based on scenario 1

In order to investigate the production capacity, received orders, sales, and lost sales during the 350 days, which is equivalent to one working year, the program was run for each medicine. Parameters considered for simulation were those calculated according to data gathered therefore the real situation is simulated based on this scenario. Table 5 embraces the simulation parameters. Figures 2-4 manifest the charts derived from simulation.

**Table 5:** The scenario 1 simulation parameters

**T = 350**					
**mx**			**nx**		
**The non-antibiotic medicine**	**The antibiotic medicine**	**The semi-solid medicine**	**The non-antibiotic medicine**	**The antibiotic medicine**	**The semi-solid medicine**
*0.005600682*	*0.003054849*	*0.0044090946*	0*.*994399318	*0.996945*	*0.995909054*

*Scenario 2*

It was decided to investigate what would happen to the production capacity of non-antibiotic medicine manufacturing agent if the parameter (mx) was decreased to half? Table 6 contains the results.

**Table 6:** The outcomes of running the simulation program based on scenario 2

**The non-antibiotic medicine**				
**T = 350 days**				
**m = 0.005600682**		**m = 0.002800341**	
**Variable name**	**The simulation result**	**Variable name**	**The simulation result**
Wn	30445.298	Wn	15222.649
NPn	5405553.042	NPn	5420775.691

*Scenario 3*

**Figure 5:**
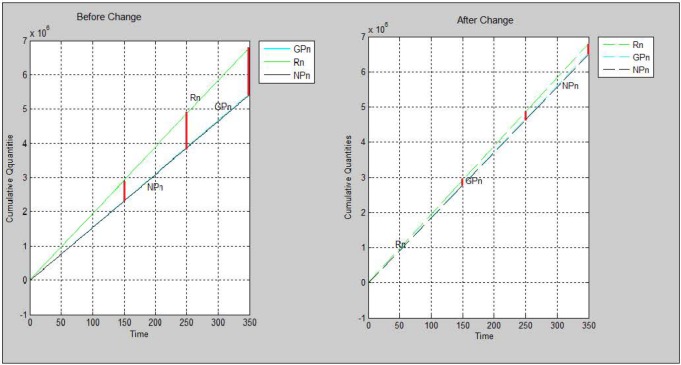
The charts resulted from running the simulation program based on scenario 1 before and after increasing production capacity

Based on a research have been done in the company by a team that is responsible to improve the processes and their performance, it was not clarified correctly how long the processes last; otherwise, the speed of production processes at some stages can be increased. Poor maintenance is another reason which has an impact on the production capacity because it causes the production line stops working. The new scenario for simulation is increasing the production capacity 1.2 times as a result of solving the noted problems. The results of simulating the non-antibiotic medicine’s supply chain under the new condition are mentioned on Table 7 and Figure 5.

**Table 7:** The outcomes of running the simulation program based on scenario 3.

**The non-antibiotic medicine**			
**T = 350 days** **m = 0.005600682**			
**Before increasing production capacity (A = 1)**		**After increasing production capacity (A = 1.2)**	
**Variable name**	**The result of simulation**	**Variable name**	**The result of simulation for T days**
GPn	5435998.34	GPn	6523198
NPn	5405553.042	Npn	6486663.65

*Scenario 4*

According to this scenario, the possibility of receiving 145000 units’ orders for the non-antibiotic medicine which should be prepared during next 70 days while the company confronts lack of raw material and the suppliers are domestic was investigated. Table 8 embraces the results.

**Table 8:** The outcomes of running the simulation program based on scenario 4.

**The Non-Antibiotic Medicine**		
**T = 70 days** **m = ** **0.005600682** **oq = 145000** **Lead time = 90**		
**GPn**	**NPn**	**Nt**
1071425.54	1065424.8263	100.56

## Discussion

According to findings, none of supply chains pertinent to each medicine are agile. all three cases face lost sales because the sale quantities are less than received orders leading to having a supply chain which is not agile due to lack of ability to respond to all demands. If we compare the agility condition for each supply chain together, the semi-solid medicine has the worse situation; the non-antibiotic medicine’s supply chain is placed in the next step and the supply chain pertained to the antibiotic medicine has the best situation. 

By reducing the portion of the waste produced daily belonging to the non-antibiotic medicine, the amount of cumulative produced waste will be decreased, and cumulative net production of this medicine will be increased. Decreasing waste leads to utilize the raw material and financial resources in the more optimized manner. In other words, the company’s resources are applied more efficiently which its advantages are to protect the environment more and more. Protecting the environment is the aim of green paradigm. Decreasing waste propels the supply chain to move toward the lean paradigm more because the main goal of this paradigm is doing more with less. 

According to scenario three the production capacity was increased by 20% while there were not any changes for other parameters and conditions. As a result, the both cumulative quantities of gross and net production were increased. As it is depicted on Figure 5, the gap between the NPn and Rn charts are decreased too. Although the supply chain is not agile yet, more order request could be fulfilled. Consequently, the company would get more benefit. 

In scenario four, the result pertained to nt is 100.5 and the simulation time is 70; in fact, nt > 70 then based on formula the order will be rejected due to lack of time.

## Conclusion

Some problems like lack of correct time assessment for manufacturing processes, or poor maintenance exist in the company. The mentioned problems cause the production capacities to be decreased. As a result, not only cannot the company respond to all order requests received by the sale agent, but it also cannot respond to demands increasing. Therefore, each selected medicines’ supply chain is not agile. As mentioned, GMP and ISO14001 are implemented in the company leading the company to have good green situation but by reducing waste, more activities can be done while less resources are applied. In this manner, the company’s supply chain can move toward being more lean and green.
